# Identification of sources and bioaccumulation pathways of MeHg in subantarctic penguins: a stable isotopic investigation

**DOI:** 10.1038/s41598-018-27079-9

**Published:** 2018-06-11

**Authors:** Marina Renedo, David Amouroux, Zoyne Pedrero, Paco Bustamante, Yves Cherel

**Affiliations:** 1 Littoral Environnement et Sociétés (LIENSs), UMR 7266 CNRS-Université de la Rochelle, 2 rue Olympe de Gouges, 17000 La Rochelle, France; 2 CNRS/UNIV PAU & PAYS ADOUR, Institut des Sciences Analytiques et de Physico-chimie pour l’Environnement et les Materiaux, UMR 5254, 64000 Pau, France; 3 Centre d’Etudes Biologiques de Chizé (CEBC), UMR 7372 du CNRS-Université de La Rochelle, 79360 Villiers-en-Bois, France

## Abstract

Seabirds are widely used as bioindicators of mercury (Hg) contamination in marine ecosystems and the investigation of their foraging strategies is of key importance to better understand methylmercury (MeHg) exposure pathways and environmental sources within the different ecosystems. Here we report stable isotopic composition for both Hg mass-dependent (e.g. δ^202^Hg) and mass-independent (e.g. Δ^199^Hg) fractionation (proxies of Hg sources and transformations), carbon (δ^13^C, proxy of foraging habitat) and nitrogen (δ^15^N, proxy of trophic position) in blood of four species of sympatric penguins breeding at the subantarctic Crozet Islands (Southern Indian Ocean). Penguins have species-specific foraging strategies, from coastal to oceanic waters and from benthic to pelagic dives, and feed on different prey. A progressive increase to heavier Hg isotopic composition (δ^202^Hg and Δ^199^Hg, respectively) was observed from benthic (1.45 ± 0.12 and 1.41 ± 0.06‰) to epipelagic (1.93 ± 0.18 and 1.77 ± 0.13‰) penguins, indicating a benthic-pelagic gradient of MeHg sources close to Crozet Islands. The relative variations of MeHg concentration, δ^202^Hg and Δ^199^Hg with pelagic penguins feeding in Polar Front circumpolar waters (1.66 ± 0.11 and 1.54 ± 0.06‰) support that different MeHg sources occur at large scales in Southern Ocean deep waters.

## Introduction

As a result of its severe toxicity, mercury (Hg) is considered as a worldwide pollutant of major concern for humans and wildlife^1^. It is present in all compartments of the Earth, transported over long distances and accumulated in the environment^2^. Anthropogenic pressure has perturbed the global Hg cycle and tripled Hg concentrations in oceanic surface waters since pre-industrial periods^3^. The elemental form of Hg (Hg^0^) from the atmosphere can be oxidized into Hg^2+^ and deposited in the surface of the ocean, where one part can be rapidly reduced back to Hg^0^. Mercury is present principally as dissolved Hg^0^ or inorganic Hg^2+^ in oceanic waters, but inorganic forms can be methylated by microbiological^4,5^ or abiotic processes^6^, leading to the incorporation of methylmercury (MeHg) in organisms and its consequent biomagnification in marine food webs. Vertical profiles of MeHg oceanic distribution^7–9^, including in the Southern Ocean^10^, showed that surface waters consistently present lower MeHg concentrations due to more rapid photodegradation, and that increasing MeHg concentrations are found with depth, peaking at low-oxygen and/or microbially active intermediate waters^8,11,12^. Consequently, an increasing gradient of total Hg (THg) concentrations was found in fish caught from the surface (epipelagic zone) to deeper waters (mesopelagic zone)^13–15^.

Seabirds are meso- to top predators within marine ecosystems and are therefore exposed to elevated concentrations of MeHg via dietary uptake. They are recognized as effective bioindicators of marine Hg contamination at different spatial scales according to their life cycle^16,17^. Most seabirds disperse or migrate from the breeding grounds during the inter-nesting period, during which they use different food and feeding ecology strategies. Flying seabirds can cover long distances at that time, thus resulting in the integration of Hg originated from both the breeding and inter-breeding foraging zones in their tissues. Therefore, the use of flying seabirds as bioindicators requires a good knowledge of their feeding ecology over the entire annual cycle to better interpret their Hg levels and exposure pathways. Compared to flying birds, the flightless diving penguins exploit relatively spatially restricted foraging zones all year long and are thus representative of Hg contamination in more limited areas, which make them interesting models for biomonitoring studies^18,19^.

Investigating several penguin species provide access to different marine environmental compartments since they have species-specific foraging ecologies. They feed on a large diversity of prey in different oceanographic ecosystems, both horizontally (from the neritic to the oceanic domains) and vertically, as they forage at different depths of the water column (from the epipelagic to the mesopelagic zones). In this study, we investigated the four sympatric penguin species that breed at the subantarctic Crozet Islands (Southern Indian Ocean): king *Aptenodytes patagonicus*, gentoo *Pygoscelis papua*, macaroni *Eudyptes chrysolophus* and eastern rockhopper *E. chrysocome filholi* penguins. The king penguin (KP) is a large oceanic species that feed on mesopelagic fish (myctophids) at deep depths (100–300 m) in distant southern foraging grounds located in the vicinity of the Polar Front^20–22^. In contrast, the medium-sized gentoo penguin (GP) is a coastal neritic species that dive both pelagically and benthically to feed opportunistically on a large diversity of prey, including swarming crustaceans and benthic fish^23,24^. The smaller and closely-related macaroni (MP) and rockhopper (RP) penguins forage in offshore waters where they primarily target swarming crustaceans (euphausiids and hyperiids) in the top 70 m of the water column^21,24–26^. Here, we measured Hg isotopes in penguin blood samples with the main objective of exploring potentially different MeHg trophic sources due to the bird contrasted foraging ecology. Blood Hg is known to reflect recent Hg exposure (over the last weeks preceding sampling) and penguins are more restricted to areas near their colonies at the time of sample collection (near the end of the breeding period). Hence, Hg isotopic composition of penguin blood samples was considered as indicative of Hg values in waters surrounding the Crozet Islands^27^.

Mercury has seven stable isotopes that undergo mass dependent fractionation (MDF, δ^202^Hg) as a result of many physical, chemical or biological processes, namely volatilization^28^, reduction^29^, methylation or demethylation^30–33^, photochemical reactions^34^, and trophic^35^ and metabolic processes^36,37^. Because of different combinations of all these processes in the environment, Hg MDF occurs with different degrees of magnitude, thus providing information about Hg processes and specific reservoirs of ecosystems. However, due the complexity of the Hg biogeochemical cycle, Hg sources cannot be easily differentiated in a given environment, especially when using top predators as bioindicators because they integrate spatio-temporally the trophic web. Moreover, photochemical reactions induce significant Hg mass independent fractionation (MIF, here Δ^199^Hg), wherein principally odd isotopes are enriched or depleted in reaction products relative to the even isotopes^34^. Recent observations also reported MIF of Hg even-mass isotopes, mainly in samples derived from the atmosphere^38^. Contrary to Hg MDF, no substantial Hg MIF has been observed during trophic processes^35,39^, meaning that MIF of odd isotopes can be used as a conservative tracer of MeHg sources in predators. This allows investigating photochemical processes before MeHg uptake in the food web^34^. Therefore, the combination of MDF with MIF is used as an effective double tracer of both Hg sources and processes in the environment.

An increasing number of studies have successfully applied Hg isotope analysis for elucidating sources and pathways of MeHg in the environment^40^. In aquatic ecosystems, Hg MIF is highly sensitive to photochemical reactions and varies as a function of the extent of light penetration at different locations or depths. For example, higher photodemethylation rates in surface waters leads to higher Hg MIF in epipelagic than in mesopelagic fish^41^. Fish MIF signature reveals that MeHg without MIF is produced at the pycnocline, thus diluting the MIF of MeHg exported from the surface mixed layer^41^. Higher magnitudes of MIF have also been observed in oceanic *versus* coastal organisms^35,42,43^. This gradient is mainly attributed to enhanced MeHg photodemethylation in oceanic waters due to higher light penetration, while higher water turbidity and benthic MeHg inputs lead to lower extent of photodemethylated Hg in coastal waters^35^. Consequently, we hypothesized that Hg MIF values in penguins should present an increasing gradient from benthic to oceanic, and from mesopelagic to epipelagic foragers.

Stable isotopes of carbon (δ^13^C) and nitrogen (δ^15^N) are used as relevant proxies of foraging habitat and trophic position of consumers, respectively. The usefulness of the stable isotope method has already been deeply investigated in the Southern Ocean, with seabird δ^13^C values indicating their latitudinal foraging grounds and depicting both offshore *versus* inshore consumers and benthic *versus* pelagic ones^22,44^. Since consumers are enriched in ^15^N over their food, δ^15^N values indicate the trophic position of consumers within a given trophic web, thus allowing making  interpretations about trophic relationships in ecological studies^45^. Thus, we used δ^13^C and δ^15^N values to help explaining the potential variations in Hg stable isotopes in penguins and to trace the origin, trophic transfer and bioaccumulation processes of Hg in marine food webs, as it was previously demonstrated in fish^41,42,44,46^.

The main objective of this work was to investigate the effectiveness of Hg stable isotope ratios in seabird tissues to discern and quantify MeHg sources and exposure pathways in the different marine compartments in which they forage. We hypothesized that contrasted ecological strategies among penguins would determine the uptake of distinct environmental MeHg sources and would lead to interspecies differences in blood Hg isotopic composition. The exploration of these Hg isotopic ratios in combination with blood δ^13^C and δ^15^N values were expected to provide new insights into the complex factors controlling Hg biogeochemical processes and help identifying the sources of MeHg ultimately accumulated in marine top predators.

## Results

### Blood Hg concentrations and Hg isotopic composition

Blood THg concentrations differed amongst penguin species (Kruskal Wallis, H = 30.09, p < 0.0001), with king (KP) and gentoo (GP) penguins presenting higher concentrations than macaroni (MP) and rockhopper (RP) penguins (Table 1). Accordingly, blood MeHg was overall different (H = 28.87, p < 0.0001), with KP and GP showing higher concentrations (both 1.9 µg g^−1^) than MP and RP (1.0 and 0.9 µg g^−1^, respectively). All individual penguins presented a large predominance of MeHg in their blood (94 ± 2%, range: 91–98%, n = 42).

**Table 1:** Food and feeding ecology, including blood δ^13^C (as a proxy of foraging habitat) and δ^15^N (as a proxy of trophic position) values, together with blood THg, MeHg and Hg isotopic composition (all isotopes and individual data in Supplementary Table S1) of subantarctic penguins from Possession Island, Crozet Archipelago (n, number of individuals).

Species	Diet	Diving	n	δ^13^C	δ^15^N	THg	MeHg	MeHg	δ^202^Hg	∆^199^Hg	∆^199^Hg/∆^201^Hg
		behaviour		(‰)	(‰)	(µg g^−1^)	(µg g^−1^)	(%)	(‰)	(‰)	ratio
King penguin	fish	mesopelagic	11	−21.8 ± 0.4^A^	10.1 ± 0.2^A^	2.01 ± 0.29^A^	1.88 ± 0.28^A^	93 ± 1	1.49 ± 0.11^A^	1.60 ± 0.04^A^	1.16 ± 0.04^A^
Gentoo penguin	crustaceans, fish	epipelagic, benthic	11	−18.6 ± 0.3^B^	8.2 ± 0.6^B^	2.04 ± 1.00^A^	1.89 ± 0.93^A^	93 ± 1	1.45 ± 0.12^A^	1.41 ± 0.06^B^	1.18 ± 0.04^A^
Macaroni penguin	crustaceans (fish)	epipelagic	10	−20.0 ± 0.7^C^	8.6 ± 0.4^B^	1.06 ± 0.16^B^	1.01 ± 0.15^B^	96 ± 2	1.66 ± 0.11^B^	1.54 ± 0.06^C^	1.14 ± 0.02^A^
Eastern rockhopper penguin	crustaceans (fish)	epipelagic	10	−20.8 ± 0.3^D^	8.6 ± 0.4^B^	0.97 ± 0.20^B^	0.93 ± 0.19^B^	95 ± 1	1.93 ± 0.18^C^	1.77 ± 0.13^D^	1.15 ± 0.04^A^

Values are means ± SD. Values not sharing the same superscript letter are statistically different.

Blood samples showed large ranges of individual δ^202^Hg (MDF) and ∆^199^Hg (MIF) values (1.28–2.12‰ and 1.31–1.95‰, respectively, n = 42). Both blood δ^202^Hg and ∆^199^Hg values differed among penguins (H = 26.94 and 32.27, respectively, both p < 0.0001), except KP and GP that showed identical δ^202^Hg values. Blood δ^202^Hg and ∆^199^Hg values increased in the order GP = KP < MP < RP and GP < MP < KP < RP, respectively (Table 1). RP notably showed high inter-individual variability in their blood ∆^199^Hg values (from 1.55 to 1.93‰).

Measured blood δ^202^Hg values followed the predicted theoretical MDF line (Supplementary Fig. S1A). In contrast, measured blood δ^199^Hg values diverged from the δ^199^Hg theoretical MDF line (Supplementary Fig. S1B), indicating that all blood samples showed MIF of the ^199^Hg (and ^201^Hg) odd isotopes. Overall penguin blood samples displayed a ∆^199^Hg/∆^201^Hg slope of 1.16 ± 0.05 (R^2^ = 0.98, p < 0.0001) (Supplementary Fig. S2). Because of the low inter-species range and high intra-species homogeneity of Hg isotopic values, the measured ∆^199^Hg/∆^201^Hg slopes for each penguin species were not accurate. Hence, we calculated the mean ∆^199^Hg/∆^201^Hg ratios for each penguin species (Table 1). Blood ∆^200^Hg values were not significantly different from zero and no statistical differences of ∆^200^Hg values were found between populations (Supplementary Fig. S1A). Therefore, no MIF of even Hg isotopes was detected in penguin blood, as already mentioned in a previous publication on avian blood and feathers^27^.

### Blood δ^13^C and δ^15^N values

**Figure 1:**
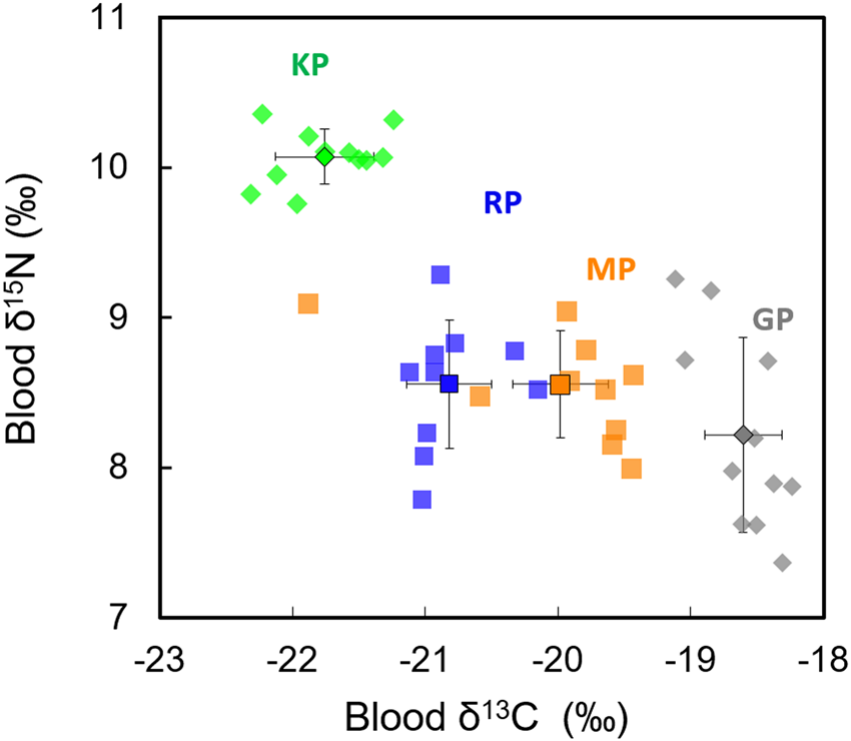
Blood δ^15^N and δ^13^C values of subantarctic penguins from Possession Island, Crozet Archipelago. Abbreviations: GP, gentoo penguin; KP, king penguin; MP, macaroni penguin; RP, rockhopper penguin.

Penguins were segregated by their δ^13^C and δ^15^N values (Fig. 1). The four penguin species presented distinct δ^13^C values (Kruskal Wallis, H = 35.30, p < 0.0001), with a progressive ^13^C enrichment from KP to GP (Table 1). Blood δ^15^N values were also different (H = 25.24, p < 0.0001). They allow splitting species into two groups, with MP, RP and GP differing from KP by their 1.5–1.8‰ lower δ^15^N values (Table 1).

### Relationship between blood Hg isotopic composition and δ^13^C and δ^15^N values

**Figure 2:**
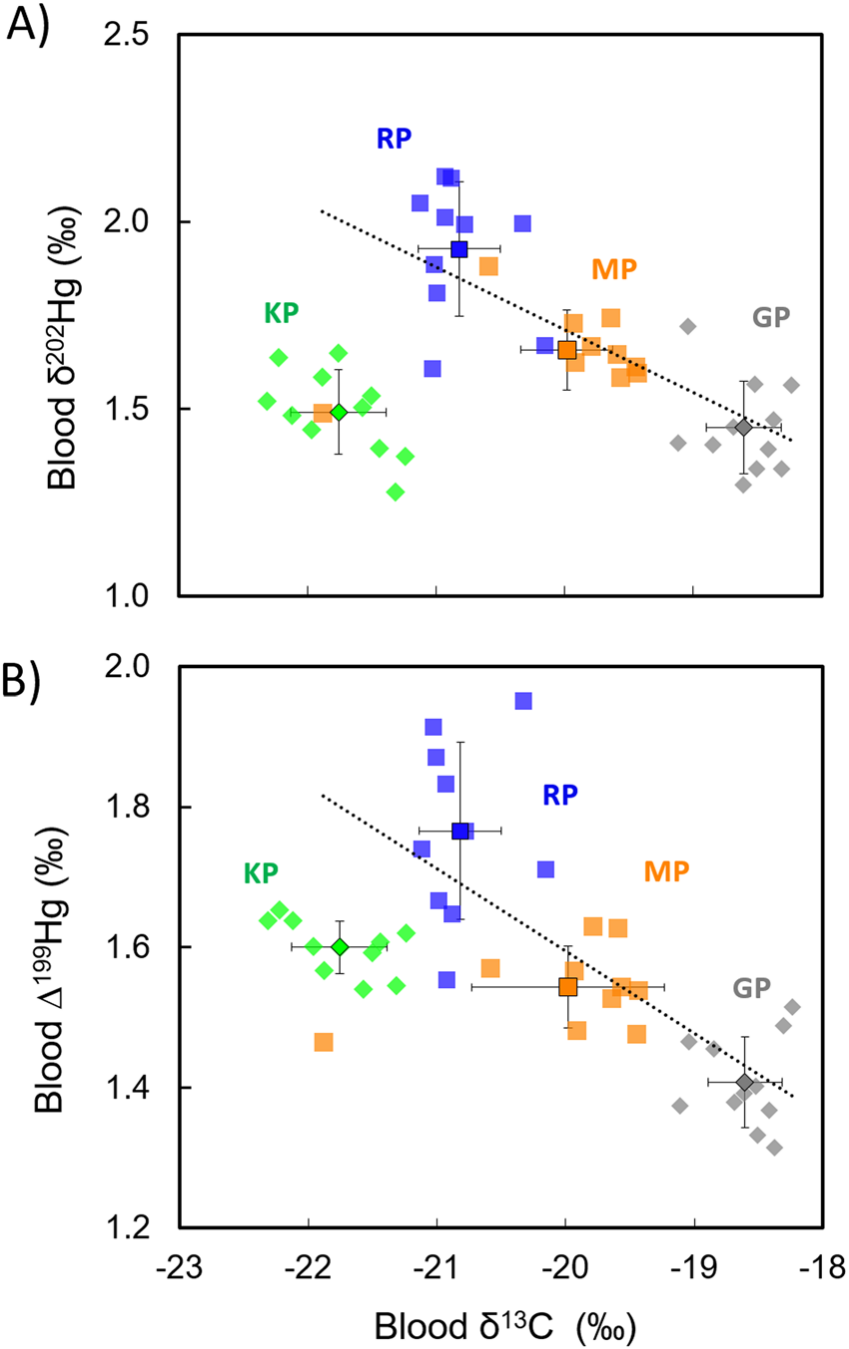
Relationship between Hg isotopes (δ^202^Hg and Δ^199^Hg) and δ^13^C values of subantarctic penguins from Possession Island, Crozet Archipelago. (**A**) Blood δ^202^Hg versus δ^13^C values. Regression equation (excluding KP) is y = −0.17x −1.64, R^2^ = −0.73, p < 0.0001. (**B**) Blood Δ^199^Hg versus δ^13^C values. Regression equation (excluding KP) is y = −0.12x −0.74, R^2^ = −0.71, p < 0.0001. Abbreviations: GP, gentoo penguin; KP, king penguin; MP, macaroni penguin; RP, rockhopper penguin.

Excluding KP values, highly negative correlations were found for δ^13^C values with δ^202^Hg values (R^2^ = −0.73, p < 0.0001) and with ∆^199^Hg values (R^2^ = −0.71, p < 0.0001) (Fig. 2). In contrast, no significant correlation was observed between blood δ^202^Hg and δ^15^N values (R^2^ = −0.10, p = 0.52) and between blood ∆^199^Hg and δ^15^N values (R^2^ = 0.03, p = 0.30) (Supplementary Fig. S2).

## Discussion

MeHg is the predominant Hg species in blood of penguins, irrespective of THg concentrations or species. Consequently, measured Hg isotopic composition of this tissue corresponds almost exclusively to MeHg. The predominance of MeHg in blood allows (i) the direct comparison of blood THg as a proxy of MeHg among the four penguin species, and (ii) the exploration of MDF and MIF values measured on THg to trace MeHg pathways in each marine compartment used by penguins. In the following sections, variations of Hg isotopic composition among penguins are discussed and interpreted as a function of their species-specific ecological characteristics in order to estimate sources and processes involving MeHg in the Crozet marine environment. We first interpreted blood δ^13^C values to define the foraging habitats of penguins and then combined δ^13^C with δ^202^Hg and Δ^199^Hg values to depict potentially different Hg sources and/or processes between the inshore and offshore environments. The relationship between blood δ^15^N values and Hg isotopes was also explored to test the potential influence of trophic processes on penguin δ^202^Hg and Δ^199^Hg values.

### Variations in foraging habitats (δ^13^C) between penguin populations

In waters surrounding subantarctic islands, δ^13^C values decrease from neritic to oceanic waters (inshore-offshore δ^13^C gradient), and from warm to cold waters (latitudinal δ^13^C gradient), thus allowing using δ^13^C values to assess the main foraging habitats of seabirds^21^. A large range of δ^13^C values was observed across penguin species with decreasing values from gentoo (GP) to king (KP) penguin. KP exhibited much lower δ^13^C values than the other two oceanic penguins (macaroni MP and rockhopper RP), which is in agreement with their well-known southern foraging grounds from Crozet Islands down to the Polar Front^21,22^. RP showed significantly lower δ^13^C values than MP, indicating that they forage at more southern latitudes than MP during the breeding period^21^. Finally, the more positive δ^13^C values of GP compared to the other penguins are in agreement with the inshore feeding habits of the species^21^. Species-specific δ^13^C values clearly demonstrate that each of the four penguins breeding at the Crozet Islands document a different compartment of the marine environment, thus allowing studying the processes affecting Hg behavior and its fate in various ecosystems from the Southern Ocean.

### Relation between Hg isotopes and penguin trophic ecology (δ^15^N)

Differences in dietary composition amongst pelagic penguins are well illustrated by δ^15^N values being higher in the fish-consumer species (KP) than in crustacean-feeders (MP and RP). KP preferentially rely on mesopelagic fish so they have a higher trophic position than MP and RP that rely mainly on swarming euphausiids and hyperiids^47^. Moreover, mesopelagic fish are known to accumulate elevated MeHg concentrations as a result of enhanced Hg methylation in deeper waters^13–15^. Consequently, both its higher trophic position and mesopelagic foraging habitat explain why the blood THg, and therefore MeHg concentrations of KP were twice higher compared to the epipelagic crustacean-eaters MP and RP. Consequently, blood δ^13^C values (Fig. 1) indicate that KP foraged in an oceanic ecosystem that is not closely connected to the Crozet archipelago, where the carbon pump is driven by the local advection and iron fertilization. The lack of correlation between blood ∆^199^Hg and δ^15^N values is consistent with the absence of MIF during trophic transfer^35,48–50^. While MDF could be produced during trophic transfer^35,37,48^, no correlation was observed between δ^202^Hg and δ^15^N values. Based on the existing knowledge of Hg isotopic fractionation dynamics, we concluded that the observed MIF (∆^199^Hg) variations between penguin species are linked to distinct MeHg sources having undergone different degrees of photochemical reactions. Since no effect of trophic level (δ^15^N) was found for δ^202^Hg and ∆^199^Hg values, the most plausible explanation of Hg isotopic variations is the consequence of species-specific foraging habitats within the marine ecosystems.

### Hg isotopic values and penguin species-specific foraging habitats

#### MIF characteristics and penguin foraging depths

Photochemical reactions of MeHg and inorganic Hg in the water column are the main drivers of Hg odd-MIF values and each process is characterized by a different ∆^199^Hg/∆^201^Hg ratio. Since this ∆^199^Hg/∆^201^Hg ratio is assumed to be preserved in the food chain after MeHg assimilation by primary producers and during biomagnification up the food web, it is used to identify mechanisms involving MIF variations^34^. Theoretical slopes have been experimentally designed in aquatic systems^34^, corresponding to 1.36 ± 0.02 for MeHg photodemethylation and 1.00 ± 0.02 for inorganic Hg photoreduction in freshwater with natural DOC. Although studies in freshwater fish also reported Δ^199^Hg/Δ^201^Hg slopes close to 1.3^48,49,51^, slightly lower Δ^199^Hg/Δ^201^Hg slopes have been observed in marine organisms^41–43,52–55^. This effect may be the result of different ligands associated with Hg in aqueous solutions, such as dissolved organic matter^34,56,57^, or dissolved cations or halogens in seawater^40^. Indeed, variable Δ^199^Hg/Δ^201^Hg slopes have been recently documented for MeHg photodemethylation under different types and concentrations of DOC^57^, indicating that low concentrations of DOC relative to MeHg could lead to lower ∆^199^Hg/∆^201^Hg slopes. The overall ∆^199^Hg/∆^201^Hg slope for penguin blood samples (1.16 ± 0.05) (Supplementary Fig. S2) is consistent with those previously reported in marine fish muscle^41,42,55^ and seabird eggs^43^. Due to the predominance of MeHg in penguin blood, and assuming a potential diminution in MIF slope due to low DOC concentrations in subantarctic waters, the obtained ratio indicates an accumulation of residual MeHg that has principally undergone photochemical demethylation.

Hg MIF in marine fish has been used to estimate the relative proportion of MeHg formed in the open ocean that is photochemically degraded prior its entry into the food web^41^. Thus, it has been shown to be an effective proxy of fish foraging depths^41^. Assuming MeHg photodemethylation as the major photochemical process, we estimated the percentage of presumed photodemetylated MeHg before entering the food web based on experimental studies^34,57^ (detailed in SI). The estimated extent of MeHg photodemethylation varied slightly between penguin species (∼13 to ∼16%), a difference that is surprisingly low when taking into account the range of habitats used by the penguins (from coastal benthic/pelagic feeders to mesopelagic feeders). Considering the existence of higher amounts of DOC in benthic waters relative to the pelagic domains, more substantial differences were expected between compartments, even for similar MeHg concentrations. The photodemethylation extent estimated in an Antarctic coastal ecosystem (∼13–18%)^58^ based on the same experimental models^57^ was similar to our findings. Slightly higher range of MeHg photodemethylation was observed in the Arctic Ocean from ice-covered to non-ice-covered marine areas (∼8–16%)^52^. Another study in fish from the Gulf of Mexico estimated that MeHg in coastal fish was ∼10–20% degraded in contrast to oceanic fish whose percentage of photodemethylated MeHg was ∼40–65%^42^. Even higher in surface waters, photodegradation appears overall limited in the Southern Indian Ocean compared to subtropical waters (e.g. Gulf of Mexico), as a consequence of lower sunlight extent and slighter angle of incidence.

Rapid light attenuation with depth leads to the inhibition of MeHg photodemethylation and thus to lower Hg odd isotopes MIF values^41^. Although such Hg MIF is much more sensitive to photochemical reactions, photodemethylation also induces MDF with the remaining MeHg enriched in heavier isotopes^34^. In the North Pacific Ocean, Blum *et al*.^41^ documented a ∆^199^Hg offset of ∼5‰ between fish feeding at the surface mixed layer and at 600 m depth. Here, we aimed to assess the correlation between Hg MIF and penguin foraging depths in the Southern Indian Ocean. A gradual increase of ∆^199^Hg values was observed from the coastal and mixed benthic/pelagic forager (GP) to the two pelagic penguins that feed in Crozet waters, with RP having higher values than MP. Although both MP and RP forage in near surface waters, the significantly different blood ∆^199^Hg values between species (∼0.20‰) suggest differences in their foraging depth intervals during the breeding period. On the other hand, KP are representative of Polar Front waters during the sampling period and they were out of this Hg MIF-foraging depth trend. Despite their mesopelagic behaviour, KP presented higher ∆^199^Hg values than epipelagic MP (mean difference 0.06‰), which is likely associated to a specific relation between Hg MIF and the characteristics of ocean waters either close to Crozet (MP) or to the Polar Front (KP).

#### Tracing distinct MeHg sources over inshore-offshore and benthic-pelagic gradients

**Figure 3:**
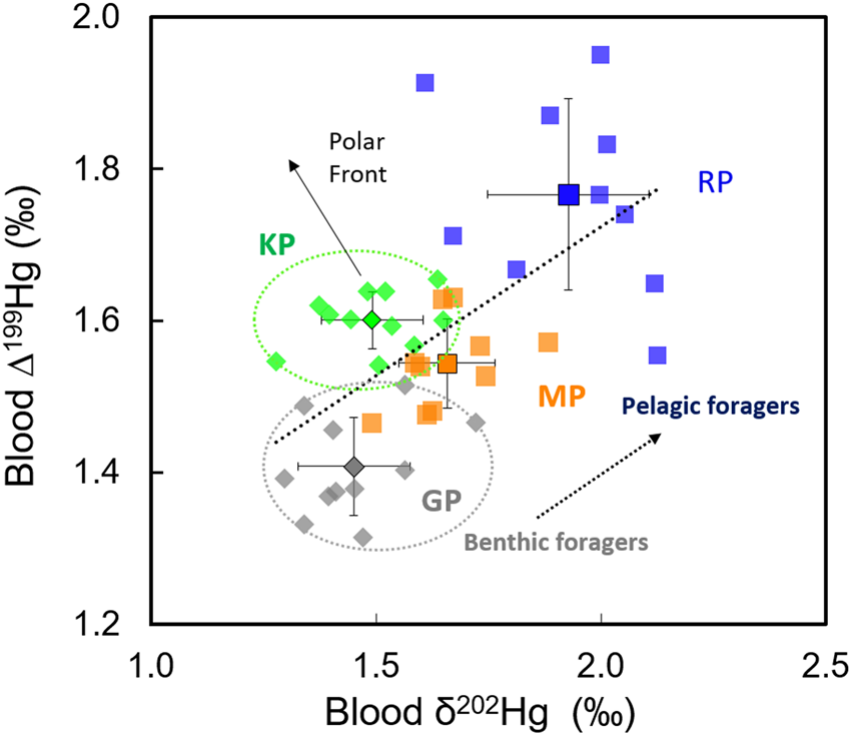
Blood Hg MIF *versus* MDF values (Δ^199^Hg *versus* δ^202^Hg) of subantarctic penguins from Possession Island, Crozet Archipelago. Regression equation is y = 0.39x + 0.93, R^2^ = 0.60, p < 0.0001. Abbreviations: GP, gentoo penguin; KP, king penguin; MP, macaroni penguin; RP, rockhopper penguin.

For a complete understanding of the exposure pathways to MeHg relative to species-specific foraging habitats in penguins, both in horizontal (inshore-offshore) and in vertical (benthic-pelagic) dimensions, we combined Hg isotopic discrimination using both MDF (δ^202^Hg) and MIF (∆^199^Hg) values. A gradual increase of both δ^202^Hg and ∆^199^Hg values was observed in the three penguin species foraging in Crozet waters, in the order GP < MP < RP (Fig. 3). The overall range of δ^202^Hg values (1.28 to 2.12‰) of penguins is slightly higher than those observed in pelagic and benthic fish from the Gulf of Mexico^42^, estuarine waters of the Labrador Sea^44^ and Hawaii coastal and marine areas^55^. However, penguin ∆^199^Hg values (1.31 to 1.95‰) fall within the range of the values observed in fish of these three mentioned regions^42,44,55^.

Experimentally determined ∆^199^Hg/δ^202^Hg ratios during aquatic MeHg photodemethylation exhibited a slope of 2.43 ± 0.10^34^ whereas microbial demethylation and reduction only affect δ^202^Hg values (∆^199^Hg/δ^202^Hg slope ~0)^29,31,33^. Previous studies have estimated the percentage of bacterial MeHg demethylation based on these experimentally obtained ∆^199^Hg/δ^202^Hg slopes assuming that only these two pathways are affecting this slope in the marine water column. For instance, similar ∆^199^Hg/δ^202^Hg ratios were observed in Hawaiian benthic fish^55^ and in pelagic and benthic fish from the Gulf of Mexico^42^, accounting for ~60% of estimated Hg biotic degradation^55^. Nevertheless, only 7% of MeHg biotic degradation was estimated in pelagic fish from the North Pacific Ocean^41^ by using this approach^55^. The overall ∆^199^Hg /δ^202^Hg ratio for penguins (0.39 ± 0.08) would suggest that bacterial degradation accounted for ~85% of total MeHg degradation before its incorporation into the food web. When excluding KP, a ∆^199^Hg /δ^202^Hg ratio of 0.50 ± 0.09 (p < 0.0001) would indicate more than 80% of MeHg demethylation by microbial processes in Crozet waters. Both a lower extent of MeHg photodemethylation and a higher influence of processes responsible of MDF such as Hg biotic transformations could explain the lower ∆^199^Hg/δ^202^Hg ratio in subantarctic and Polar Front waters when compared to other marine ecosystems.

Benthic-pelagic gradient of MeHg sources in Crozet Islands: Previous studies have already documented Hg isotopic differences between coastal and oceanic marine organisms^35,42,43^, indicating the existence of contrasted environmental MeHg sources with distinct Hg isotopic baselines and different extent of aquatic photochemistry. Significantly lower δ^202^Hg and ∆^199^Hg values exhibited by benthic/pelagic GP relative to pelagic foragers is indicative of their higher accumulation of Hg with a sediment origin. Sediment Hg isotopic composition is characterized by (i) a different Hg isotopic baseline if compared to oceanic waters (ii) close to zero MIF and negative MDF extent and (iii) further no or low photochemical reactions of benthic MeHg. Therefore, lighter Hg isotopic values are typically found in benthic coastal biota relative to oceanic organisms as a result of a higher continental influence^42,43^. No sediment Hg isotopic data are available from the Crozet Islands, but it is likely that δ^202^Hg values are negative and ∆^199^Hg values are close to zero, as commonly observed in sediments from other sites such as in the Arctic Ocean (δ^202^Hg: −1.37 ± 0.38‰; ∆^199^Hg: −0.02 ± 0.07‰)^59^ and the Antarctic coasts (δ^202^Hg: −0.39 ± 0.49‰; ∆^199^Hg: 0.71 ± 0.43‰)^58^. Indeed, Zheng *et al*.^58^ observed similar MIF values between historical sediment profiles and penguin and seal fresh faeces, suggesting that faeces were the dominant sources of Hg to the sediments at different time periods. Due to the huge penguin (and other seabirds) populations, a significant fraction of Hg accumulated in coastal sediments from the Crozet Islands could be of ornithogenic origin, thus showing similar isotopic values as other Antarctic sediments^58^. In coastal ecosystems, a higher turbidity reduces light penetration, thus limiting Hg photochemistry. This phenomenon, together with the influence of benthic Hg inputs, may also contribute to the lower ∆^199^Hg values of blood MeHg in the coastal GP relative to MP and RP that feed in more offshore waters.

The significant correlations between blood δ^13^C and Hg isotopic values (Fig. 2) suggest a progressive transition from terrestrial to marine values along both a horizontal (inshore-offshore) and a vertical (benthic-pelagic) gradient. This is in agreement with *in situ* Hg methylation in sediment and reduced exposure to sunlight (due to turbidity and/or depth) as the main mechanisms lowering Hg isotopic values in benthic ecosystems. Meanwhile, a gradual increase in ∆^199^Hg (and δ^202^Hg) was observed from inshore to offshore waters as a consequence of higher magnitude of photochemical processes in more opened areas. Nevertheless, the low variations in Hg isotopes values between benthic and pelagic penguins compared to previous inshore-offshore values measured in other marine ecosystems^42,43^ seems to indicate a higher degree of mixing between benthic and pelagic MeHg sources. The remote location of the Crozet Islands, which are surrounded by deep oceanic waters (4000–5000 m), could explain a lower impact of the sediment-derived MeHg inputs compared to continental coastal zones. Moreover, these islands have a plateau of around 150 km wide that interacts with different water masses derived from the Antarctic Circumpolar Current^60^, thus potentially favouring the recirculation and mixing of MeHg from different sources. Indeed, the relatively low range of δ^13^C values and the similar δ^202^Hg/∆^199^Hg ratio of the three penguins seem to be coherent with common environmental MeHg production sources, with the resulting MeHg accumulating either in the benthic or pelagic food webs in Crozet waters. MeHg in offshore marine ecosystems likely derives primarily from inorganic Hg deposited from the atmosphere^61^. However, the high degree of oceanic recirculation within the water column in the vicinity of Crozet Islands could favour the redistribution of MeHg between the surface to deeper zones of the water column and its combination with the MeHg originating from the benthic zones.

**Figure 4:**
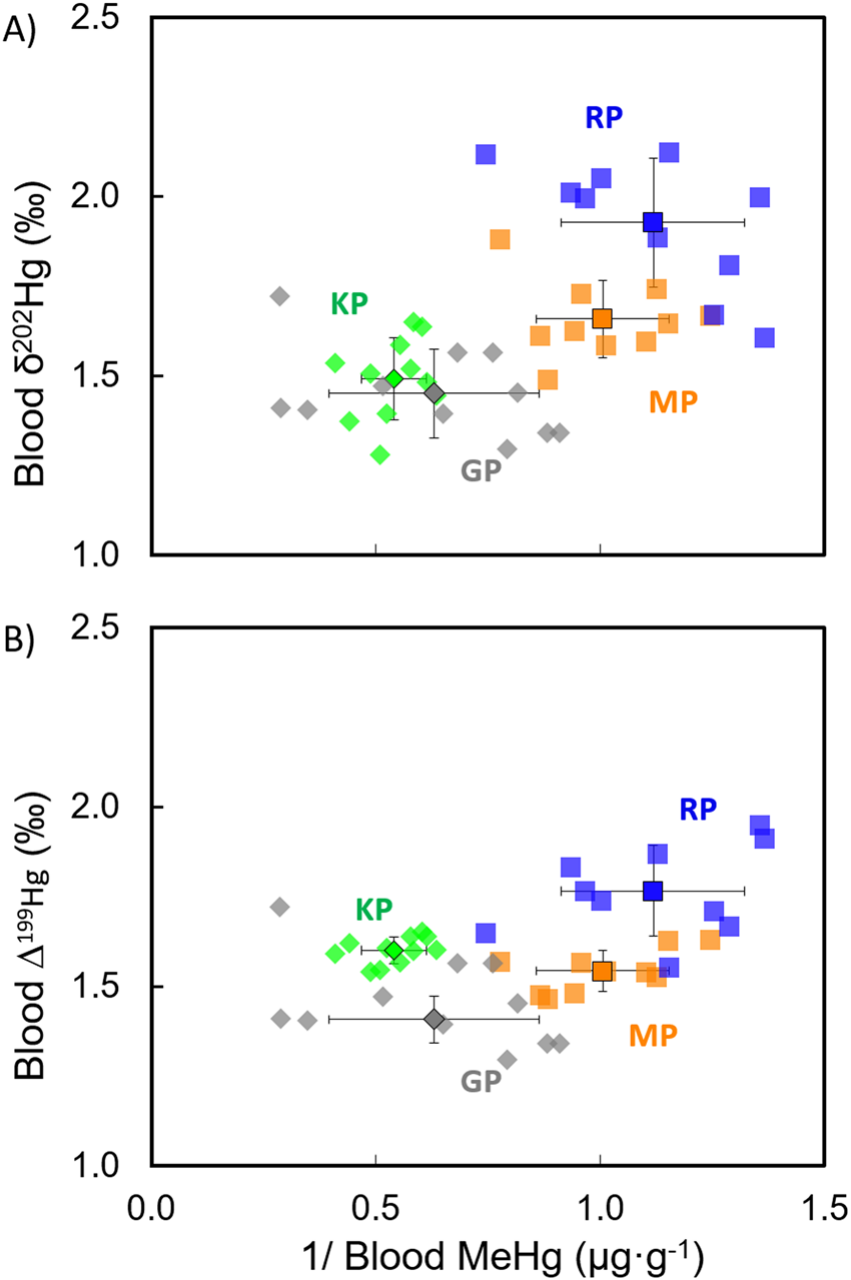
Relationships between Hg isotopes (δ^202^Hg and Δ^199^Hg) and MeHg concentration values. (**A**) Blood δ^202^Hg and inverse of MeHg concentration, and (**B**) blood Δ^199^Hg and inverse of MeHg concentration of subantarctic penguins from Possession Island, Crozet Archipelago. Abbreviations: GP, gentoo penguin; KP, king penguin; MP, macaroni penguin; RP, rockhopper penguin.

Different pelagic MeHg sources between Crozet and distant (Polar Front) waters: The significant trend between MeHg concentrations and both δ^202^Hg and ∆^199^Hg values (Fig. 4) clearly illustrates a common mixing source for the four subantarctic penguins with a dominant sediment MeHg source in benthic/pelagic penguins with higher Hg levels (GP) and a more diluted pelagic MeHg source with lower concentrations at the sea surface (RP). Significantly higher MeHg concentrations in KP, as discussed above, could also be associated to their higher trophic level compared to MP and RP. However, the lower Hg isotopic values of KP suggest that they feed on a distinct food web that is MeHg-enriched compared to the pelagic ecosystem close by the Crozet Islands. Both seasonal release of nutrients and summer algal bloom^62,63^ lead to higher primary productivity in Polar Front waters, which favours bacterial activity compared to northern waters^64^. Therefore, greater methylation yields at the Polar Front can also contribute to the higher MeHg concentrations accumulated in KP compared to MP and RP. Moreover, algal blooms may lead to higher accumulation and availability of organic matter close to the photic zone of the water column^63^. These conditions probably favour both microbial production of MeHg at shallower depth^11,12^ and its higher photodemethylation under exposure to sunlight. This hypothetical effect should provide higher MIF signatures in MeHg accumulated in mesopelagic food webs of the Polar Front.

In conclusion, RP, MP and GP seem to be connected to the same dominant benthic Hg source, which appears to be associated to the Crozet shelf sediments diluted and mixed in nearby waters with a pelagic source. On the other hand, Hg stable isotope values indicate that KP is relying on a different and unique Hg isotopic baseline due to specific inorganic Hg source and methylation/demethylation pathways in such offshore open waters.

Measuring Hg isotopic composition in sympatric penguins of the Crozet Islands allows demonstrating that penguins are exposed to different levels and sources of MeHg when they forage in three distinct food webs (i.e. coastal benthic, pelagic in Crozet waters and mesopelagic at the Polar Front). A clear pelagic-benthic gradient and the influence of the Polar Front productive waters were found. The combination of blood isotopic values (δ^202^Hg, Δ^199^Hg, δ^13^C and δ^15^N) of penguins together with the documented information about their feeding ecology allows characterizing the major factors explaining their levels of MeHg exposure in relationship with their contrasted ecological habits. The observed Hg isotopic variations from benthic to pelagic penguins suggest the existence of sources mixing deriving from MeHg production at depth and increased photochemical processes when going to shallower offshore waters. The study pinpoints the need of further investigations to depict the various biogeochemical sources of MeHg and the processes leading to accumulation of MeHg in biota from these most remote ecosystems on Earth.

## Material and Methods

All experimental protocols were approved by the French Polar Institute IPEV ethic committee and the methods were carried out in accordance with the approved guidelines and regulations.

### Study site and sampling procedure

Sample collection was conducted during the austral summer 2011–2012 (from October to February; Supplementary Table S1) at Ile de la Possession (46°26′S, 51°45′E), Crozet Islands, which are located within the Subantarctic Zone. Ten to 11 randomly-chosen breeding adults were blood sampled at the end of the chick-rearing period by venepuncture of a flipper vein using heparinized syringes. Whole blood was centrifuged to separate plasma from blood cells (hereafter blood). Blood samples were kept frozen at −20 °C until Hg and isotopic analyses in France.

### Sample preparation and analytical methods

#### Hg speciation and Hg isotopic analyses

For Hg speciation analyses, Hg was extracted from blood samples (0.10–0.15 g) by alkaline microwave digestion with 5 mL of tetramethylammonium hydroxide (25% TMAH in H_2_O, Sigma Aldrich)^65^. Hg species analyses were carried out by GC-ICPMS Trace Ultra GC equipped with a Triplus RSH autosampler coupled to an ICP-MS XSeries II (Thermo Scientific, USA) in the laboratory IPREM (Pau, France). Details of the extraction method, analysis and quantification of Hg species are included in the SI and are further detailed elsewhere^27^. Blood total Hg concentration was also quantified by using an advanced Hg analyzer (AMA-254, Altec) for the intercomparison with total Hg concentrations obtained by Hg speciation analyses, i.e. the sum of inorganic and organic Hg.

Prior to Hg isotopic analyses, blood samples (0.05–0.10 g) were digested with 3 or 5 mL of HNO_3_ acid (65%, INSTRA quality) after a predigestion step overnight at room temperature and later extraction in Hotblock at 75 °C during 8 h (6 h in HNO_3_, plus 2 h after the addition of 1/3 of the total volume of H_2_O_2_ (30%, ULTREX quality)). Hg isotopic composition was determined using cold-vapor generator (CVG)-MC-ICPMS (Nu Instruments), as detailed previously^35^. Hg isotopic values were reported as delta notation, calculated relative to the bracketing standard NIST SRM-3133 reference material to allow inter-laboratory comparisons, as described in SI. NIST SRM-997 thallium standard solution was used for the instrumental mass-bias correction using the exponential law (details of calculation in SI). Secondary standard NIST RM-8160 (previously UM-Almadén standard) was used for validation of the analytical session (Supplementary Table S2). Details of Hg isotopic composition analyses are included in the SI and are further detailed elsewhere^27^.

For the validation of the analytical results, four certified reference material were analysed: human hair IAEA-086 and NIES-13, tuna fish ERM-CE-464 and dogfish liver DOLT-4. An internal reference sample was prepared with pooled samples collected from different individuals of king penguins from the Crozet Islands (RBC-KP, red blood cells). It was analysed at each analytical session. Analytical uncertainty for delta values was calculated using SD typical errors for reference materials (Supplementary Table S2), as recommended by reference publications for standard reporting of Hg isotopic ratio uncertainties^66,67^.

#### Carbon and nitrogen stable isotopes analyses

Blood samples were freeze-dried and powdered, and subsamples were weighed with a microbalance and packed in tin containers. Carbon (δ^13^C) and nitrogen (δ^15^N) stable isotope ratios were determined in red blood cells with a continuous flow mass spectrometer (Thermo Scientific Delta V Advantage) coupled to an elemental analyser (Thermo Scientific Flash EA 1112) in the laboratory LIENSs (La Rochelle, France) (aliquots mass: ~0.3 mg). Results are in delta notation relative to Vienna PeeDee Belemnite and atmospheric N_2_ for δ^13^C and δ^15^N, respectively. Replicate measurements of internal laboratory standards (acetanilide) indicated measurement errors <0.15‰ for both δ^13^C and δ^15^N values.

### Statistical analyses

Statistical tests were performed using R 3.3.2 (RStudio)^68^. Before analyses, data were checked for normality of distribution and homogeneity of variances using Shapiro–Wilk and Breusch-Pagan tests, respectively. Since data groups did not meet specificities of normality and homoscedasticity, non-parametrical tests (Kruskal–Wallis with Conover-Iman test) were performed. Statistically significant results were set at α = 0.05. Values are means ± SD. We examined the correlations between MeHg concentrations, δ^13^C, δ^15^N and both Hg MDF (δ^202^Hg) and MIF (∆^199^Hg) using linear regressions and Pearson correlation rank tests. Hg MIF ∆^199^Hg and ∆^201^Hg values were regressed to determine if MIF ratio was consistent with photochemical degradation of MeHg and in good agreement with previous studies on marine organisms (e.g.^42,43,55^).

## Electronic supplementary material

Supplementay Information
