# Supplementary material for: Identification of sources and bioaccumulation pathways of MeHg in subantarctic penguins: a stable isotopic investigation
Source: Sci Rep. 2018 Jun 11;8:8865. doi: 10.1038/s41598-018-27079-9 (PMC5995893; doi:10.1038/s41598-018-27079-9)
Supplement: Supplementary file 1 — Supplementay Information [file 41598_2018_27079_MOESM1_ESM.pdf]

Supplementary information

Identification of sources and bioaccumulation pathways of MeHg in subantarctic penguins: a stable isotopic investigation

Marina Renedo[1,2*], David Amouroux[2], Zoyne Pedrero[2], Paco Bustamante[1], Yves Cherel[3*]

[1] Littoral Environnement et Sociétés (LIENSs), UMR 7266 CNRS-Université de la Rochelle, 2 rue Olympe de Gouges, 17000 La Rochelle, France

[2] CNRS/ UNIV PAU & PAYS ADOUR, Institut des Sciences Analytiques et de Physico-chimie pour l'Environnement et les Materiaux , UMR5254, 64000, Pau, France

[3] Centre d'Etudes Biologiques de Chizé (CEBC), UMR 7372 CNRS-Université de La Rochelle, 79360 Villiers-en-Bois, France

Corresponding authors: marina.renedoelizalde@univ-pau.fr, yves.cherel@cebc.cnrs.fr

Content: 2 tables, 3 figures

Sample preparation and analytical methods

Certified reference materials and total Hg concentration. Due to the absence of bird blood certified reference materials for Hg and MeHg concentrations, an internal reference sample was prepared with pooled samples collected from different individuals of king penguin (KP) from Crozet Islands: RBC-KP (red blood cells). This reference samples was analyzed at each analytical session. For the validation of the results, tuna fish CRM (ERM-CE-464) was additionally analyzed. Blood total Hg concentration was also quantified by using an advanced Hg analyzer (AMA-254, Altec), as described in a previous study [1], thus allowing the intercomparison with Hg total concentrations obtained by Hg speciation analyses, i.e. the sum of inorganic and organic Hg.

Hg speciation analyses. Hg was extracted from blood samples (0.10-0.15 g) with 5 mL of tetramethylammonium hydroxide (25% TMAH in H_2O, Sigma Aldrich) by microwave digestion (Discover SP-D, CEM Corporation) coupled to an autosampler Explorer 4872 96 (USA). Hg species analyses were carried out by GC-ICPMS by species-specific isotope dilution technique. Further details of the analysis and quantification of Hg species are included in a previous publication [2].

Hg isotopic analyses. Blood samples (0.05-0.10 g) were digested with 3 or 5 mL of HNO_3 acid (65%, INSTRA quality) after a predigestion step overnight at room temperature. Hotblock mineralization was carried out in Savillex vessels at 75ºC during 8 h (6 h in HNO_3 and then 2 more h after addition of 1/3 of the total volume of H_2O_2 (30%, ULTREX quality)). Hg isotopic composition was determined using cold-vapor generator (CVG)-MC-ICPMS (Nu Instruments), as detailed previously [3]. Hg isotopic values were reported as delta notation,

calculated relative to the bracketing standard NIST SRM-3133 reference material to allow inter-laboratory comparisons (See equations for delta notation below). NIST SRM-997 thallium standard solution was used for the instrumental mass-bias correction using the exponential law. Secondary standard NIST RM-8160 (previously UM-Almadén standard) was used for validation of the analytical session.

Delta notation for Hg isotopic results

The theoretical mass-dependent kinetic isotope fractionation law is reported by the $^{202}Hg/^{198}Hg$ ratio (i.e., $\delta^{202}Hg$) to predict the theoretical MDF values for $\delta^{199}Hg$, $\delta^{200}Hg$, $\delta^{201}Hg$, and $\delta^{204}Hg$. [4]. Hg isotopic compositions of samples are reported using delta notation according to the following equations:

$$\delta^{xxx}Hg = [(^{xxx/198}Hg_{sample} / ^{xxx/198}Hg_{NIST\ 3133}) - 1)] \cdot 1000\ (‰) \qquad (1)$$

Hg MIF is then reported as the deviation in isotope ratios from the theoretical variation in isotope ratios imparted by this predicted mass-dependent kinetic isotope fractionation. MIF signatures are expressed using capital delta notation, as suggested elsewhere [5], according to the following equations:

$$\Delta^{204}Hg = \delta^{204}Hg - (1.493 \cdot \delta^{202}Hg) \qquad (2)$$

$$\Delta^{201}Hg = \delta^{201}Hg - (0.752 \cdot \delta^{202}Hg) \qquad (3)$$

$$\Delta^{200}Hg = \delta^{200}Hg - (0.502 \cdot \delta^{202}Hg) \qquad (4)$$

$$\Delta^{199}Hg = \delta^{199}Hg - (0.252 \cdot \delta^{202}Hg) \qquad (5)$$

Estimation of the fraction of photodemethylated MeHg for each penguin population

The extent of photodemethylation undergone by MeHg before its incorporation into the aquatic food web was estimated by following Bergquist and Blum reference model (Bergquist and Blum, 2007). MIF fractionation factors ($\alpha_{\Delta199Hg}$ and $\alpha_{\Delta201Hg}$) were determined experimentally as a function of MeHg/DOC ratio by Rayleigh-fractionation equations in waters with the presence of 2 mg/L and 20 mg/L DOM. This same model was later extrapolated to environmental conditions in marine medium taking into account different conditions of MeHg/DOC in the Arctic Ocean (Point et al. 2011). In a more recent study by Chandan et al., (2015) photodemethylation extent was evaluated at different ratios between MeHg and reduced sulfur content in DOC (MeHg: Sred-DOC). In our study, we calculated MeHg:DOC according to previous data on the subantarctic zone of the Southern Ocean (Canario et al., 2017; Tremblay et al., 2015), MeHg amount in the pelagic zone (100-200 m depth) which is approximatively 0.48 ng per mg of dissolved organic carbon. Concentrations of DOC in surface waters were not considered since photochemical reactions are known to photodegrade organic matter and that MeHg is produced under the photic zone, where phytoplankton is degraded by bacteria. We calculated by extrapolation the MIF fractionation factors for the Crozet Islands to obtain fitting equations based on Chandan calculations (low concentrations of DOC) (Chandan et al., 2015). MeHg accumulated in GP was 13.1±0.6% photodegraded, whereas slightly greater extent of MeHg photodegradation was observed in pelagic penguins: 14.7±0.3% (KP), 14.3±0.5% (MP) and 16.2±1.1% (RP).

Supplementary Table S1. Individual blood total Hg, Hg species concentrations and C, N and Hg isotopic composition of subantarctic penguins from Possession Island, Crozet archipelago (n, number of individuals).

**Penguin species**	**Sampling date**	**Individual**	**δ^{13}C** ‰	**δ^{15}N** ‰	**MeHg** (µg g^{-1})	**iHg** (µg g^{-1})	**THg** (µg g^{-1})	**δ^{204}Hg** ‰	**δ^{202}Hg** ‰	**δ^{201}Hg** ‰	**δ^{200}Hg** ‰	**δ^{199}Hg** ‰	**Δ^{204}Hg** ‰	**Δ^{201}Hg** ‰	**Δ^{200}Hg** ‰	**Δ^{199}Hg** ‰
King penguin	Oct 2011	KPA01	-21.76	10.10	1.71	0.14	1.85	2.51	1.65	2.60	0.81	2.02	0.05	1.36	-0.02	1.60
King penguin	Oct 2011	KPA02	-21.57	10.10	2.04	0.14	2.19	2.12	1.51	2.47	0.70	1.92	-0.13	1.34	-0.05	1.54
King penguin	Oct 2011	KPA03	-22.12	9.95	1.63	0.11	1.73	2.24	1.48	2.49	0.71	2.01	0.02	1.38	-0.03	1.64
King penguin	Oct 2011	KPA04	-22.31	9.82	1.73	0.13	1.87	2.29	1.52	2.52	0.79	2.02	0.02	1.37	0.03	1.64
King penguin	Oct 2011	KPA05	-21.32	10.07	1.96	0.16	2.12	1.85	1.28	2.37	0.61	1.87	-0.06	1.40	-0.03	1.55
King penguin	Oct 2011	KPA06	-21.88	10.21	1.80	0.10	1.90	2.17	1.59	2.60	0.76	1.97	-0.20	1.41	-0.03	1.57
King penguin	Oct 2011	KPA07	-21.50	10.05	2.44	0.16	2.60	2.14	1.53	2.50	0.78	1.98	-0.16	1.35	0.01	1.59
King penguin	Oct 2011	KPA08	-21.96	9.76	1.57	0.12	1.70	2.12	1.45	2.50	0.71	1.97	-0.04	1.41	-0.02	1.60
King penguin	Oct 2011	KPA09	-21.44	10.05	1.91	0.12	2.02	2.12	1.40	2.38	0.71	1.96	0.03	1.33	0.00	1.61
King penguin	Oct 2011	KPA10	-21.24	10.32	2.26	0.15	2.42	2.10	1.37	2.45	0.69	1.97	0.05	1.41	0.00	1.62
King penguin	Oct 2011	KPA11	-22.22	10.35	1.65	0.11	1.77	2.33	1.64	2.61	0.77	2.07	-0.12	1.38	-0.06	1.65
Gentoo penguin	Oct 2011	GPA01	8.72	3.21	3.50	0.29	3.79	2.57	1.72	2.52	0.83	1.90	0.00	1.23	-0.03	1.47
Gentoo penguin	Oct 2011	GPA02	7.89	3.18	1.94	0.15	2.09	2.22	1.47	2.28	0.74	1.68	0.02	1.17	0.00	1.31

Gentoo penguin	Oct 2011	GPA03	7.87	3.18	1.32	0.09	1.40	2.35	1.56	2.46	0.76	1.91	0.01	1.28	-0.03	1.52
Gentoo penguin	Oct 2011	GPA04	9.26	3.22	3.47	0.24	3.71	2.16	1.41	2.28	0.74	1.73	0.06	1.22	0.03	1.37
Gentoo penguin	Oct 2011	GPA05	7.98	3.23	1.23	0.10	1.33	2.24	1.45	2.29	0.74	1.75	0.08	1.20	0.01	1.38
Gentoo penguin	Oct 2011	GPA06	7.37	3.19	1.13	0.11	1.25	1.96	1.34	2.23	0.65	1.83	-0.04	1.23	-0.03	1.49
Gentoo penguin	Oct 2011	GPA07	8.19	3.24	1.47	0.11	1.58	2.37	1.57	2.35	0.79	1.80	0.04	1.17	0.00	1.40
Gentoo penguin	Oct 2011	GPA08	8.71	3.19	1.54	0.11	1.64	2.13	1.39	2.25	0.65	1.72	0.05	1.20	-0.05	1.37
Gentoo penguin	Oct 2011	GPA09	7.62	3.22	1.10	0.10	1.19	2.03	1.34	2.14	0.67	1.67	0.03	1.13	-0.01	1.33
Gentoo penguin	Oct 2011	GPA10	9.18	3.19	2.87	0.18	3.06	2.15	1.40	2.20	0.71	1.81	0.05	1.15	0.00	1.46
Gentoo penguin	Oct 2011	GPA11	7.62	3.20	1.26	0.10	1.36	1.98	1.30	2.18	0.60	1.72	0.04	1.21	-0.05	1.39
Macaroni penguin	Jan 2012	MPA11	8.16	3.21	0.87	0.08	0.95	2.48	1.65	2.65	0.82	2.04	0.02	1.41	0.00	1.63
Macaroni penguin	Jan 2012	MPA12	9.04	3.22	1.05	0.04	1.09	2.59	1.73	2.69	0.90	2.00	0.01	1.39	0.03	1.57
Macaroni penguin	Jan 2012	MPA13	8.58	3.26	1.06	0.04	1.10	2.51	1.63	2.53	0.80	1.89	0.08	1.31	-0.02	1.48
Macaroni penguin	Jan 2012	MPA14	8.79	3.26	0.80	0.02	0.82	2.49	1.67	2.64	0.86	2.05	0.00	1.38	0.02	1.63
Macaroni penguin	Jan 2012	MPA15	8.62	3.23	0.91	0.04	0.95	2.31	1.60	2.52	0.81	1.94	-0.08	1.32	0.01	1.54
Macaroni penguin	Jan 2012	MPA16	8.52	3.26	0.89	0.03	0.92	2.61	1.74	2.63	0.90	1.97	0.00	1.32	0.03	1.53
Macaroni penguin	Jan 2012	MPA17	8.26	3.30	0.99	0.04	1.03	2.45	1.59	2.53	0.81	1.94	0.08	1.34	0.01	1.54
Macaroni penguin	Jan 2012	MPA18	8.00	3.25	1.16	0.09	1.25	2.41	1.61	2.55	0.72	1.88	0.01	1.34	-0.09	1.48
Macaroni penguin	Jan 2012	MPA19	9.10	3.24	1.13	0.04	1.18	2.23	1.49	2.40	0.71	1.84	0.01	1.28	-0.04	1.47

Macaroni penguin	Jan 2012	MPA20	8.48	3.29	1.29	0.06	1.34	2.80	1.88	2.83	0.95	2.05	-0.01	1.41	0.00	1.57
Eastern rockhopper penguin	Feb 2012	RPA11	8.53	3.18	0.80	0.05	0.84	2.57	1.67	2.72	0.89	2.13	0.07	1.46	0.05	1.71
Eastern rockhopper penguin	Feb 2012	RPA12	8.78	3.22	0.74	0.04	0.78	2.92	2.00	3.13	1.00	2.45	-0.06	1.63	0.00	1.95
Eastern rockhopper penguin	Feb 2012	RPA13	8.84	3.20	1.04	0.08	1.12	3.04	2.00	3.06	1.05	2.27	0.06	1.56	0.05	1.77
Eastern rockhopper penguin	Feb 2012	RPA14	8.75	3.19	1.07	0.05	1.13	3.00	2.01	3.08	1.05	2.34	-0.01	1.57	0.04	1.83
Eastern rockhopper penguin	Feb 2012	RPA15	8.08	3.19	0.89	0.05	0.93	2.74	1.89	2.99	0.96	2.35	-0.08	1.57	0.01	1.87
Eastern rockhopper penguin	Feb 2012	RPA16	9.29	3.19	1.34	0.05	1.39	3.22	2.12	3.05	1.08	2.18	0.06	1.46	0.01	1.65
Eastern rockhopper penguin	Feb 2012	RPA17	8.64	3.20	1.00	0.03	1.03	3.04	2.05	3.09	1.03	2.26	-0.02	1.54	0.00	1.74
Eastern rockhopper penguin	Feb 2012	RPA18	8.64	3.22	0.87	0.03	0.89	3.10	2.12	3.05	0.93	2.09	-0.07	1.45	-0.13	1.55
Eastern rockhopper penguin	Feb 2012	RPA19	8.23	3.19	0.78	0.03	0.81	2.78	1.81	2.79	0.89	2.12	0.08	1.42	-0.02	1.67
Eastern rockhopper penguin	Feb 2012	RPA20	7.79	3.19	0.73	0.04	0.77	2.45	1.61	2.90	0.93	2.32	0.05	1.69	0.12	1.91

Supplementary Table S2. Mean values (± 2SD) of Hg isotopic composition obtained for reference materials: UM-Almadén, IAEA-086 and NIES-13 (human hair), ERM-CE-464 (tuna fish), DOLT-4 (dogfish liver) and RBC-KP (IRM penguin red blood cells).

Sample	Reference	n	$\delta^{204}Hg$ ‰	$\delta^{202}Hg$ ‰	$\delta^{201}Hg$ ‰	$\delta^{200}Hg$ ‰	$\delta^{199}Hg$ ‰	$\Delta^{204}Hg$ ‰	$\Delta^{201}Hg$ ‰	$\Delta^{200}Hg$ ‰	$\Delta^{199}Hg$ ‰
NIST RM 8610	This study	38	-0.84±0.22	-0.54±0.14	-0.45±0.14	-0.27±0.418	-0.17±0.14	-0.03±0.13	-0.04±0.08	0.00±0.14	-0.03±0.12
	Reference values		-0.82±0.07	-0.56±0.03	-0.46±0.02	-0.27±0.01	-0.17±0.01	-	-0.04±0.01	0.00±0.01	-0.03±0.02
IAEA-086	This study	6	0.94±0.25	0.73±0.13	0.77±0.09	0.37±0.11	0.44±0.16	-0.14±0.26	0.22±0.08	0.00±0.09	0.26±0.15
	Yamakawa et al., 2016[10]	3	0.87±0.12	0.58±0.09	0.64±0.09	0.31±0.04	0.41±0.02	0.00±0.04	0.20±0.03	0.02±0.04	0.26±0.02
NIES-13	This study	8	2.99±0.22	2.08±0.15	3.07±0.16	1.09±0.10	2.34±0.10	-0.12±0.28	1.51±0.06	0.04±0.04	1.81±0.09
	Yamakawa et al., 2016[10]	11	2.76±0.16	1.89±0.10	2.77±0.10	0.98±0.08	2.13±0.07	-0.04±0.11	1.36±0.07	0.04±0.04	1.65±0.06
ERM-CE-464	This study	2	0.91±0.13	0.64±0.04	2.37±0.03	0.40±0.01	2.49±0.01	-0.04±0.19	1.89±0.05	0.08±0.01	2.33±0.01
	Cransveld et al., 2017[10]	4	0.91±0.43	0.69±0.23	2.32±0.41	0.44±0.17	2.39±0.35	-	1.80±0.24	0.10±0.07	2.22±0.29
DOLT-4	This study	3	-0.50±0.08	-0.35±0.18	0.65±0.09	-0.16±0.05	1.00±0.09	0.02±0.02	0.92±0.18	0.01±0.04	1.09±0.08
	Cransveld et al., 2017[11]	7	-0.39±0.34	-0.22±0.18	0.67±0.18	-0.09±0.16	0.95±0.15	-	0.84±0.16	0.03±0.09	1.01±0.12
RBC-KP	This study	5	2.00±0.18	1.32±0.12	2.28±0.14	0.64±0.14	1.79±0.18	0.04±0.10	1.29±0.09	-0.02±0.09	1.46±0.16

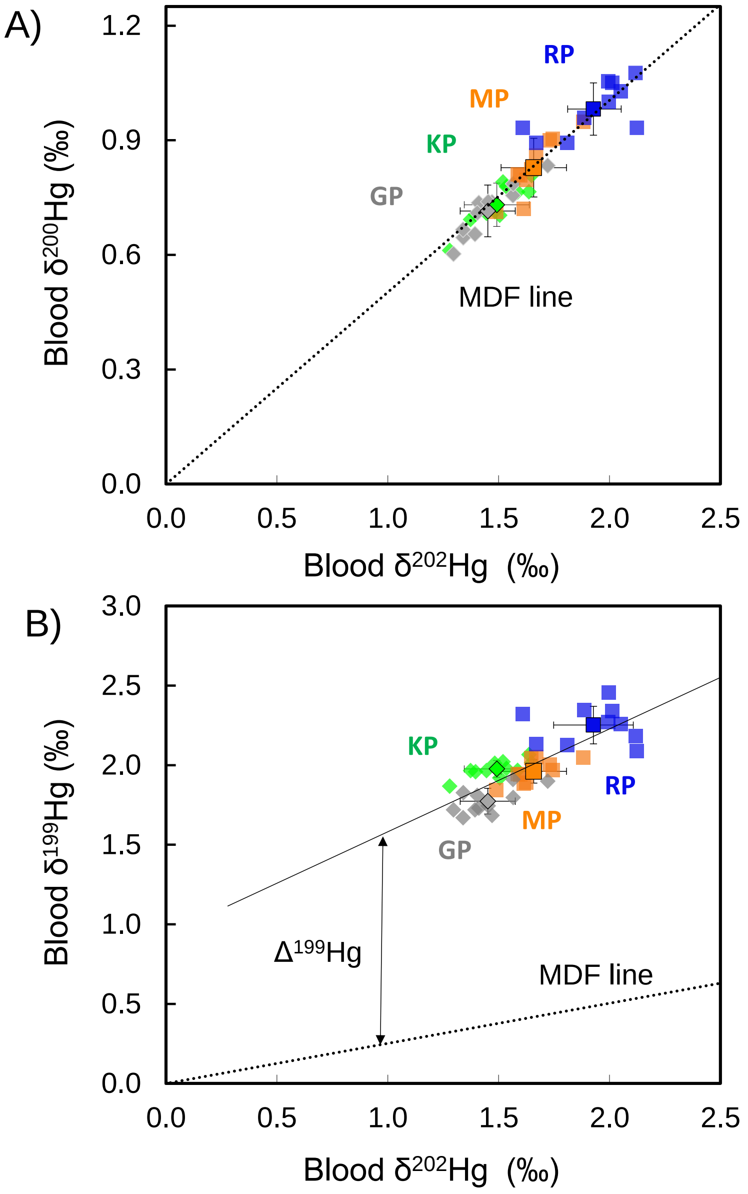

Supplementary Figure S1. Blood Hg MDF and MIF values of subantarctic penguins from Possession Island, Crozet Archipelago **A)** blood δ^{200}Hg *versus* δ^{202}Hg values, and **B)** blood δ^{199}Hg *versus* δ^{202}Hg values. Dotted lines represent the theoretically predicted MDF based on δ^{202}Hg. Abbreviations: GP, gentoo penguin; KP, king penguin; MP, macaroni penguin; RP, rockhopper penguin.

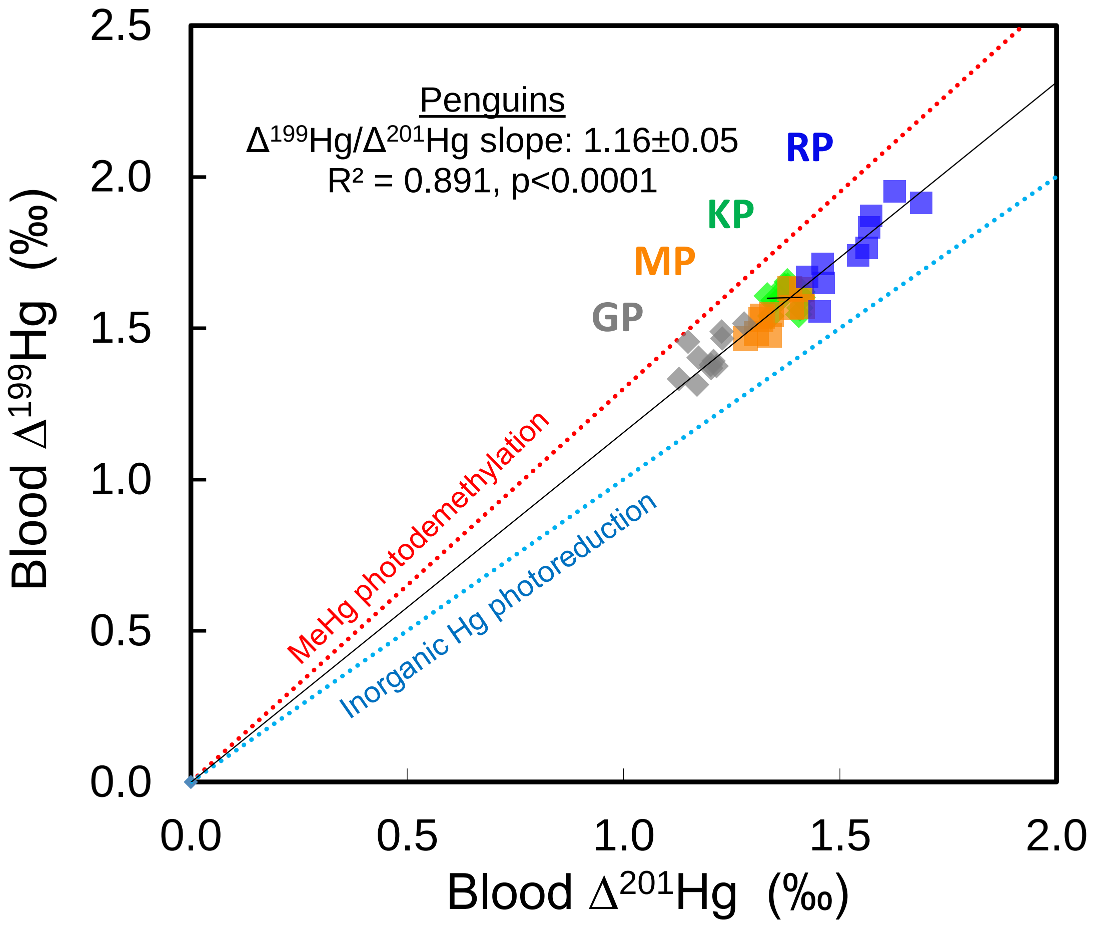

Supplementary Figure S2. Penguin blood Hg MIF values (Δ^{199}Hg *versus* Δ^{201}Hg). The solid line represents the Δ^{199}Hg/Δ^{201}Hg slope of the samples (taking intercept at origin). The red dashed line represents the theoretical Δ^{199}Hg/Δ^{201}Hg slope for MeHg photodemethylation in the water column, and the blue dashed line represents the theoretical Δ^{199}Hg/Δ^{201}Hg slope expected for photoreduction of inorganic Hg in the water column [5]. Abbreviations: GP, gentoo penguin; KP, king penguin; MP, macaroni penguin; RP, rockhopper penguin.

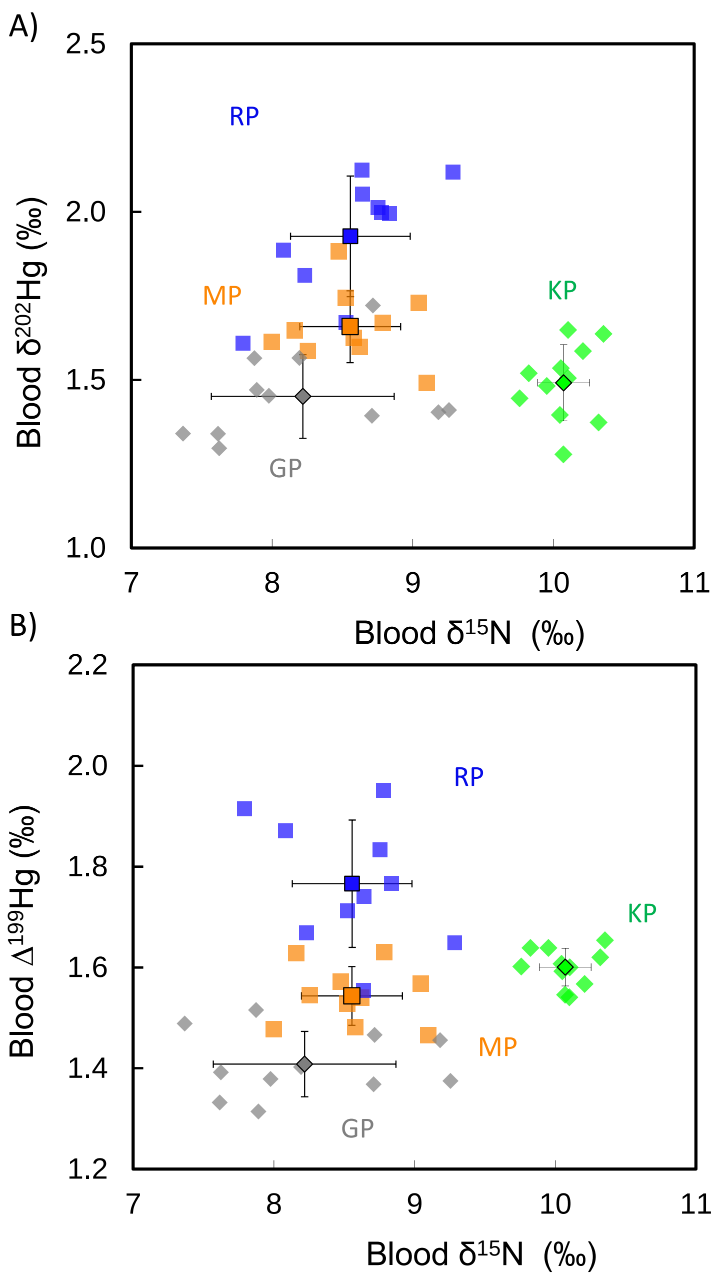

Supplementary Figure S3. A) Blood Hg MDF *versus* $\delta^{15}N$ values, and B) blood Hg MIF *versus* $\delta^{15}N$ values of subantarctic penguins from Possession Island, Crozet Archipelago. Abbreviations: GP, gentoo penguin; KP, king penguin; MP, macaroni penguin; RP, rockhopper penguin.

References

1. Carravieri, A. *et al.* From Antarctica to the subtropics : Contrasted geographical concentrations of selenium , mercury , and persistent organic pollutants in skua chicks (Catharacta spp .). *Environ. Pollut.* **228,** 464–473 (2017).
2. Renedo, M. *et al.* Assessment of mercury speciation in feathers using species-specific isotope dilution analysis. *Talanta* **174,** 100–110 (2017).
3. Perrot, V. *et al.* Higher mass-independent isotope fractionation of methylmercury in the pelagic food web of Lake Baikal (Russia). *Environ. Sci. Technol.* **46,** 5902–11 (2012).
4. Blum, J. D. & Bergquist, B. a. Reporting of variations in the natural isotopic composition of mercury. *Anal. Bioanal. Chem.* **388,** 353–359 (2007).
5. Bergquist, B. A. & Blum, J. D. Mass-dependent and -independent fractionation of Hg isotopes by photoreduction in aquatic systems. *Science* **318,** 417–20 (2007).
6. Point, D. *et al.* Methylmercury photodegradation influenced by sea-ice cover in Arctic marine ecosystems. *Nat. Geosci.* **4,** 1–7 (2011).
7. Chandan, P., Ghosh, S. & Bergquist, B. A. Mercury isotope fractionation during aqueous photoreduction of monomethylmercury in the presence of dissolved organic matter. *Environ. Sci. Technol.* **49,** 259–267 (2015).
8. Canario, J., Santos-Echeandía, J., Koch, B. P. & Laglera, L. Mercury and methylmercury in the Atlantic sector of the Southern Ocean. *Deep. Res. Part II* **138,** 52–62 (2017).
9. Tremblay, L., Caparros, J., Leblanc, K. & Obernosterer, I. Origin and fate of particulate and dissolved organic matter in a naturally iron-fertilized region of the Southern Ocean. *Biogeosciences* **12,** 607–621 (2015).
10. Yamakawa, A., Takeuchi, A., Shibata, Y., Berail, S. & Donard, O. F. X. Determination of Hg isotopic compositions in certified reference material NIES No. 13 Human Hair by cold vapor generation multi-collector inductively coupled plasma mass spectrometry. *Accredit. Qual. Assur.* (2016).
11. Cransveld, A. A. E. *et al.* Mercury stable isotopes discriminate different populations of European seabass and trace potential Hg sources around Europe. *Environ. Sci. Technol.* **51,** 12219–12228 (2017).
